# Synthesis of novel bioactive lactose-derived oligosaccharides by microbial glycoside hydrolases

**DOI:** 10.1111/1751-7915.12124

**Published:** 2014-04-01

**Authors:** Marina Díez-Municio, Miguel Herrero, Agustín Olano, F Javier Moreno

**Affiliations:** Instituto de Investigación en Ciencias de la Alimentación, CIAL (CSIC-UAM), CEI (UAM+CSIC)C/ Nicolás Cabrera 9, Madrid, 28049, Spain

## Abstract

Prebiotic oligosaccharides are increasingly demanded within the Food Science domain because of the interesting healthy properties that these compounds may induce to the organism, thanks to their beneficial intestinal microbiota growth promotion ability. In this regard, the development of new efficient, convenient and affordable methods to obtain this class of compounds might expand even further their use as functional ingredients. This review presents an overview on the most recent interesting approaches to synthesize lactose-derived oligosaccharides with potential prebiotic activity paying special focus on the microbial glycoside hydrolases that can be effectively employed to obtain these prebiotic compounds. The most notable advantages of using lactose-derived carbohydrates such as lactosucrose, galactooligosaccharides from lactulose, lactulosucrose and 2-α-glucosyl-lactose are also described and commented.

## Introduction

Since Gibson and Roberfroid introduced the concept of prebiotics as non-digestible oligosaccharides that reach the colon without being hydrolyzed and are selectively metabolized by health-positive bacteria such as bifidobacteria and lactobacilli (Gibson and Roberfroid, 1995), there is a growing consumer interest in functional foods containing this class of compounds. Given that they contribute to enhance the growth of the beneficial intestinal microbiota and prevent the spread of pathogenic microorganisms, a number of functional foods based on the presence of prebiotic carbohydrates have been introduced into the market.

The prebiotic properties of oligosaccharides are determined by its monosaccharide composition and their glycosidic linkages (Sanz *et al*., 2005). Thus, the search for new prebiotic carbohydrates with improved properties has attracted great interest in academic and industrial food research. Today, there are a wide range of emerging potential prebiotics demanding robust data from human studies to be included in the world prebiotic market along with lactulose and galactooligosaccharides (GOS), both obtained from lactose, being two of the most widely used in the food and pharmaceutical industries.

Whey permeate, a waste product of the cheese industry obtained by ultrafiltration of cheese whey, contains most of the lactose originally present in milk and has a high biological oxygen demand (BOD), thereby depleting the dissolved oxygen content of the receiving water. Given the large quantity of cheese annually produced worldwide (OECD-FAO, 2013), the use of whey permeate as raw material for the production of valuable lactose derivatives is a challenge for research and industry.

In general, enzymatic processes have a great potential in the food carbohydrate field, as they normally exhibit high yields, substrate specificity, regio- and stereospecificity (Buchholz and Seibel, 2008). Oligosaccharides are normally formed from mono- and/or disaccharides that participate in glycosyl transfer reactions catalyzed by specific enzymes. For their application in foodstuffs, both the initial substrates and enzymes must have a generally recognized as safe status, reasonable cost and be easily available. Lactose and sucrose buffered solutions, as well as dairy and sugar by-products containing these disaccharides are suitable starting materials for the synthesis of functional oligosaccharides.

Enzymes devoted to the synthesis of oligosaccharides can be obtained from a wide variety of sources such as microorganisms, plants and animals. However, microbial enzymes are normally preferred for industrial production as they exhibit a number of important advantages such as (i) easier handling, (ii) higher multiplication rate, (iii) higher production yields (greater catalytic activity), (iv) genes encoding for microbial enzymes are efficiently translated and expressed as active proteins in bacteria such as *Escherichia coli*, (v) economic feasibility (i.e. fermentation for microbial expression systems are carried out on inexpensive media), (vi) enhanced versatility on acceptor substrates, (vii) better stability and (viii) regular supply due to absence of seasonal fluctuations, among others (Panesar *et al*., 2006; Filice and Marciello, 2013; Gurung *et al*., 2013). Consequently, an important number of microbial enzymes produced by fungi, bacteria or yeasts have been employed for the efficient synthesis of a wide range of dietary bioactive oligosaccharides as it is summarized in Table 1.

**Table 1:** Microbial glycoside hydrolase enzymes most frequently used for the synthesis of food bioactive oligosaccharides

Synthesized Oligosaccharide	General structure^a^ [main linkages]	Involved enzymes (EC number)	Enzyme Source (microorganism)	GH family^b^	Sugar substrate (reaction type)	References
Fructo-oligosaccharides	(Fru)n-Glc [β(2→1), β(2→6)]	β-Fructofuranosidase (EC 3.2.1.26) Inulosucrase (EC 2.4.1.9) Levansucrase (EC 2.4.1.10)	Fungi (*Aspergillus niger; A. japonicus; A. oryzae; Aureobasidium pullulans; Penicillium citrinum*) Bacteria (*Bacillus macerans*; *Zymomonas mobilis; Lactobacillus reuteri; Arthrobacter sp.*)	32, 68, 100	Sucrose (transfructosylation)	Sangeetha *et al*., 2005
Galacto-oligosaccharides	(Gal)n-Glc (Gal)n-Gal [β(1→3), β(1→4), β(1→6)]	β-Galactosidase (EC 3.2.1.23)	Fungi (*A. oryzae; A. niger; A. aculeatus*) Bacteria (*Bacillus sp.; Streptococcus thermophilus; Lactobacillus acidophilus; L. reuteri; Bifidobacterium sp.*) Yeast (*Kluyveromyces lactis; K. marxianus; Saccharomyces fragilis; Cryptococcus laurentii*)	1, 2, 3, 35, 42, 50	Lactose (transgalactosylation)	Panesar *et al*., 2006
						Torres *et al*., 2010
Galacto-oligosaccharides derived from lactulose	(Gal)n-Fru (Gal)n-Gal (Gal)n-Fru-Gal [β(1→1), β(1→4), β(1→6)]	β-Galactosidase (EC 3.2.1.23)	Fungi (*A. oryzae; A. aculeatus*) Yeast (*K. lactis*)	1, 2, 3, 35, 42, 50	Lactulose (transgalactosylation)	Cardelle-Cobas *et al*., 2008
						Martínez-Villaluenga *et al*., 2008b
Lactosucrose	β-Gal-(1→4)-α-Glc-(1→2)-β-Fru	β-Fructofuranosidase (EC 3.2.1.26) Levansucrase (EC 2.4.1.10) or β-Galactosidase (EC 3.2.1.23)	Bacteria (*Arthrobacter sp.* K-1; *Z. mobilis; Bacillus subtilis; B. natto; B. circulans*)	32, 68, 100 or 1, 2, 3, 35, 42, 50	Lactose and sucrose (transfructosylation of lactose or transgalactosylation of sucrose)	Takahama *et al*., 1991
						Pilgrim *et al*., 2001
						Park *et al*., 2005
						Han *et al*., 2009
						Li *et al*., 2009
Lactulosucrose	β-Gal-(1→4)-β-Fru-(2→1)-α-Glc	Dextransucrase (EC 2.4.1.5)	Bacteria (*Leuconostoc mesenteroides*)	70	Lactulose and sucrose (transglucosylation)	Díez-Municio *et al*., 2012a
2-α-glucosyl-lactose	β-Gal-(1→4)-α-Glc-(2→1)-α-Glc	Dextransucrase (EC 2.4.1.5)	Bacteria (*L. mesenteroides*)	70	Lactose and sucrose (transglucosylation)	Díez-Municio *et al*., 2012b
Isomalto-oligosaccharides	(Glc)n [α(1→6)]	A) α-Amylase (EC 3.2.1.1) and Pullulanase (EC 3.2.1.41) B) α-Glucosidase (EC 3.2.1.20)	Fungi (*Aspergillus sp.; Aureobasidium pullulans*) Bacteria (*Bacillus subtilis; B. licheniformis; B. stearothermophilus*)	A) 13, 14, 57, 119 B) 4, 13, 31, 63, 97, 122	Starch [hydrolysis (A) and transglucosylation (B)]	Casci and Rastall, 2006
						Goffin *et al*., 2011
Gluco-oligosaccharides	(Glc)n [α(1→2), α(1→3), α(1→4), α(1→6)].	Dextransucrase (EC 2.4.1.5)	Bacteria (*L. mesenteroides; L. citreum*)	70	Maltose and sucrose (transglucosylation)	Remaud *et al*., 1992
						Chung and Day, 2002
						Kim *et al*., 2014
Gentio-oligosaccharides	(Glc)n [β(1→6)]	Glucan endo-1,6-β-glucosidase (EC 3.2.1.75) or β-Glucosidase (EC 3.2.1.21)	Fungi (*Penicillium multicolor; A. oryzae*)	5, 30 or 1, 3, 5, 9, 30, 116	Pustulan (hydrolysis) or Gentibiose (transglucosylation)	Fujimoto *et al*., 2009
						Qin *et al*., 2011
Pectic-oligosaccharides	(GalA)n [α(1→4)] (GalA-Rha)n [α(1→2), α(1→4)] * GalA residues can be partially esterified and Rha units ramified.	Polygalacturonase (EC 3.2.1.15) Rhamnogalacturonan galacturonohydrolase (EC 3.2.1.173) Rhamnogalacturonan rhamnohydrolase (EC 3.2.1.174)	Fungi (*Fusarium moniliforme; Aspergillus pulverulentus; A. aculeatus; Kluyveromyces fragilis*) Bacteria (*B. licheniformis*)	28	Pectin (hydrolysis)	Spiro *et al*., 1993
						Olano-Martin *et al*., 2001
						Coenen *et al*., 2008
						Holck *et al*., 2011
Xylo-oligosaccharides	(Xyl)n [β(1→4)]	Endo-1,4-β-xylanase (EC 3.2.1.8) β-Xylosidase (EC 3.2.1.37)	Fungi (*Trichoderma* sp*.; A. oryzae*)	5, 8, 9, 10, 11, 12, 16, 26, 30, 43, 44, 51, 62	Xylan (hydrolysis)	Casci and Rastall, 2006
Maltosyl-fructosides	(Fru)n-Malt [β(2→1), β(2→6)]	Levansucrase (EC 2.4.1.10) Inulosucrase (EC 2.4.1.9)	Bacteria (*B. subtilis; Lactobacillus gasseri)*	32, 68	Maltose and sucrose (transfructosylation)	Canedo *et al*., (1999)
						Díez-Municio *et al*., (2013b)

a. Glc, glucose; Fru, fructose; Gal, galactose; GalA, galacturonic acid; Rha, rhamnose; Xyl, xylose; Malt, maltose.

b. According to CAZy database.

This review will firstly deal with the classification and structural features of the main microbial enzymes involved in the synthesis of food bioactive oligosaccharides, as well as the insights into the catalytic mechanism. Then, it will focus on a series of recently synthesized lactose-derived oligosaccharides, such as GOS derived from lactulose, lactulosucrose or 2-α-glucosyl-lactose, whose characterized chemical structures point out their utility as emerging bioactive food ingredients.

## Classification of microbial enzymes involved in the synthesis of food bioactive oligosaccharides

### International Union of Biochemistry and Molecular Biology (IUBMB) enzyme nomenclature

There are different systems used to classify enzymes. Concretely, enzymes involved in the synthesis of food bioactive oligosaccharides have been traditionally classified as glycosidases and glycosyltransferases (GTs) according to the recommendations of the IUBMB, which is based mostly on substrate specificity and is expressed in the Enzyme Commission (EC) number for a given enzyme (http://www.chem.qmul.ac.uk/iubmb/enzyme/). Consequently, glycosidases, i.e. enzymes that hydrolyze *O*- and *S*-glycosyl compounds, are given the code 3.2.1.x, where × represents the substrate specificity and, occasionally, also the molecular mechanism or type of glycosidic linkage (Henrissat and Davies, 1997). GTs (EC 2.4) are capable of transferring glycosyl groups, from one compound (known as donor), to water or another compound (acceptor) catalyzing, thus, oligosaccharide synthesis (Monsan and Paul, 1995). This type of enzymes are further subdivided, according to the nature of the sugar residue being transferred, into hexosyltransferases (EC 2.4.1), pentosyltransferases (EC 2.4.2), and those transferring other glycosyl groups (EC 2.4.99) (Plou *et al*., 2002). This classification system has several limitations, such as EC numbers cannot accommodate enzymes that act on several distinct substrates or do not reflect the intrinsic structural and mechanistic features of the enzymes (Coutinho *et al*., 2003).

Consequently, GTs can be also classified according to the nature of the donor molecule into three main mechanistic groups: (i) Leloir-type GTs, which require sugar nucleotides derivatives [monosaccharide (di)phosphonucleotides], (ii) non-Leloir GTs that utilize non-nucleotide donors, which may be sugar-1-phosphates, sugar-1-pyrophosphates, polyprenol phosphates or polyprenol pyrophosphates and (iii) transglycosidases, which employ non-activated sugars such as sucrose, lactose or starch (Plou *et al*., 2007). Within transglycosidases, glycansucrases are a class of microbial enzymes that polymerize either the fructosyl (i.e. fructansucrases) or the glucosyl (i.e. glucansucrases) moiety of sucrose to give β-D-fructan- or α-D-glucan-type polysaccharides. Interestingly, this type of enzymes has also the ability to transfer fructosyl or glucosyl units to suitable acceptor molecules to yield dietary oligosaccharides, and, consequently, are considered as attractive synthetic tools (André *et al*., 2010; Côté, 2011). Dextransucrases (EC 2.4.1.5) or alternansucrases (2.4.1.140) and inulosucrases (EC 2.4.1.9) or levansucrases (EC 2.4.1.10) are among the most-used microbial glucansucrases and fructansucrases, respectively, in oligosaccharide synthesis from sucrose (van Hijum *et al*., 2006).

### Carbohydrate-active enzymes database (CAZy)

In 1991, Henrissat (1991) introduced a classification of glycosidases [glycoside hydrolases (GHs)] based upon amino acid sequence similarities. This classification was settled on the principle that sequence and structure are related and, hence, useful structural and mechanistic information could be inferred from this classification system (Henrissat and Davies, 1997). That initial classification has evolved to the CAZy database (http://www.cazy.org), which describes the families of structurally related catalytic and carbohydrate-binding modules (CBMs; or functional domains) of enzymes that degrade, modify or create glycosidic bonds (Lombard *et al*., 2014). Currently, CAZy covers more than 300 protein families divided in Glycoside hydrolases (GHs, hydrolysis and/or rearrangement of glycosidic bonds), GTs (formation of glycosidic bonds), Polysaccharide lyases (non-hydrolytic cleavage of glycosidic bonds), Carbohydrate esterases (hydrolysis of carbohydrate esters), auxiliary activities [redox enzymes that act in conjunction with carbohydrate-active en*zy*mes (CAZymes)], as well as CBMs (adhesion to carbohydrates).

It is noteworthy to remark that transglycosidases and glycosidases are structurally, mechanistically and evolutionarily related and, therefore, they are included in the GH family in the CAZy classification (Coutinho *et al*., 2003). In consequence, according to this classification, GHs comprise, up to now, 133 families converging upon similar active sites for the cleavage of glycosidic linkages. From now on, we will focus on this type of enzymes given its relevance in the synthesis of food bioactive oligosaccharides (Table 1).

## Structural and catalytic insights into microbial GHs used for the synthesis of food bioactive oligosaccharides

### Three-dimensional structure determination of GHs

Three-dimensional structural studies are essential to reveal the identity of important amino acid residues involved in catalysis and to elucidate the mechanism in the enzymatic synthesis of oligosaccharides. It is largely known that the substrate specificity and the mode of action of GHs are driven by details of their three-dimensional structures rather than by their global fold (Davies and Henrissat, 1995). In this sense, structural analyses and site-directed mutagenesis studies carried out in GHs have revealed that linkage specificity is controlled by the topology of only a few key amino acids located at the enzyme active site (Irague *et al*., 2013).

Regarding microbial β-galactosidases, 13 crystal structures have been elucidated to date and included in the Protein Data Bank (PDB; http://www.rcsb.org). Table 2 shows the PDB accession code (PDB ID) and the GH family of these enzymes. The β-galactosidases from *Kluyveromyces lactis* and *Aspergillus oryzae* are commercially available and they are the most frequently used in food industry for the treatment of milk and whey (Pereira-Rodríguez *et al*., 2012; Maksimainen *et al*., 2013). Additionally, as it will be explained below in more detail, these enzymes are also employed for the efficient synthesis of oligosaccharides by transgalactosylation of lactose and lactulose (Table 1).

**Table 2:** Microbial β-galactosidases and glycansucrases whose three-dimensional structures have been elucidated and included in the Protein Data Bank

Microbial enzyme source	PDB ID	GH family	Reference
β-Galactosidases			
*Sulfolobus solfataricus*	1UWR	1	Gloster *et al*., 2004
*Escherichia coli*	1DP0	2	Juers *et al*., 2000
*Arthrobacter* sp. C2-2	1YQ2	2	Skalova *et al*., 2005
*Bacteroides fragilis*	3FN9	2	Unpublished
*Kluyveromyces lactis*	3OB8	2	Pereira-Rodríguez *et al*., 2012
*Penicillium* sp.	1TG7	35	Rojas *et al*., 2004
*Trichoderma reesei*	3OG2	35	Maksimainen *et al*., 2011
*Caulobacter crescentus*	3U7V	35	Unpublished
*Bacteroides thetaiotaomicron*	3D3A	35	Unpublished
*Streptococcus pneumoniae*	4E8C	35	Cheng *et al*., 2012
*Aspergillus oryzae*	4IUG	35	Maksimainen *et al*., 2013
*Thermus* sp. A4	1KWG	42	Hidaka *et al*., 2002
*Bacillus circulans* sp. *alkalophilus*	3TTS	42	Maksimainen *et al*., 2012
Glycansucrases			
Fructansucrases			
*Aspergillus japonicus*	3LIH	32	Chuankhayan *et al*., 2010
*Bacillus subtilis*	1PT2	68	Meng and Fütterer, 2003
*Bacillus megaterium*	3OM2	68	Strube *et al*., 2011
*Gluconacetobacter diazotrophicus*	1W18	68	Martínez-Fleites *et al*., 2005
*Lactobacillus johnsonii* NCC 533	2YFR	68	Pijning *et al*., 2011
Glucansucrases			
*Lactobacillus reuteri* 180	3KLK	70	Vujičić-Žagar *et al*., 2010
*Lactobacillus reuteri* 121	4AMC	70	Pijning *et al*., 2012
*Streptococcus mutans*	3AIE	70	Ito *et al*., 2011
*Leuconostoc mesenteroides* NRRL B-1299	3TTQ	70	Brison *et al*., 2012

**Fig. 1:**
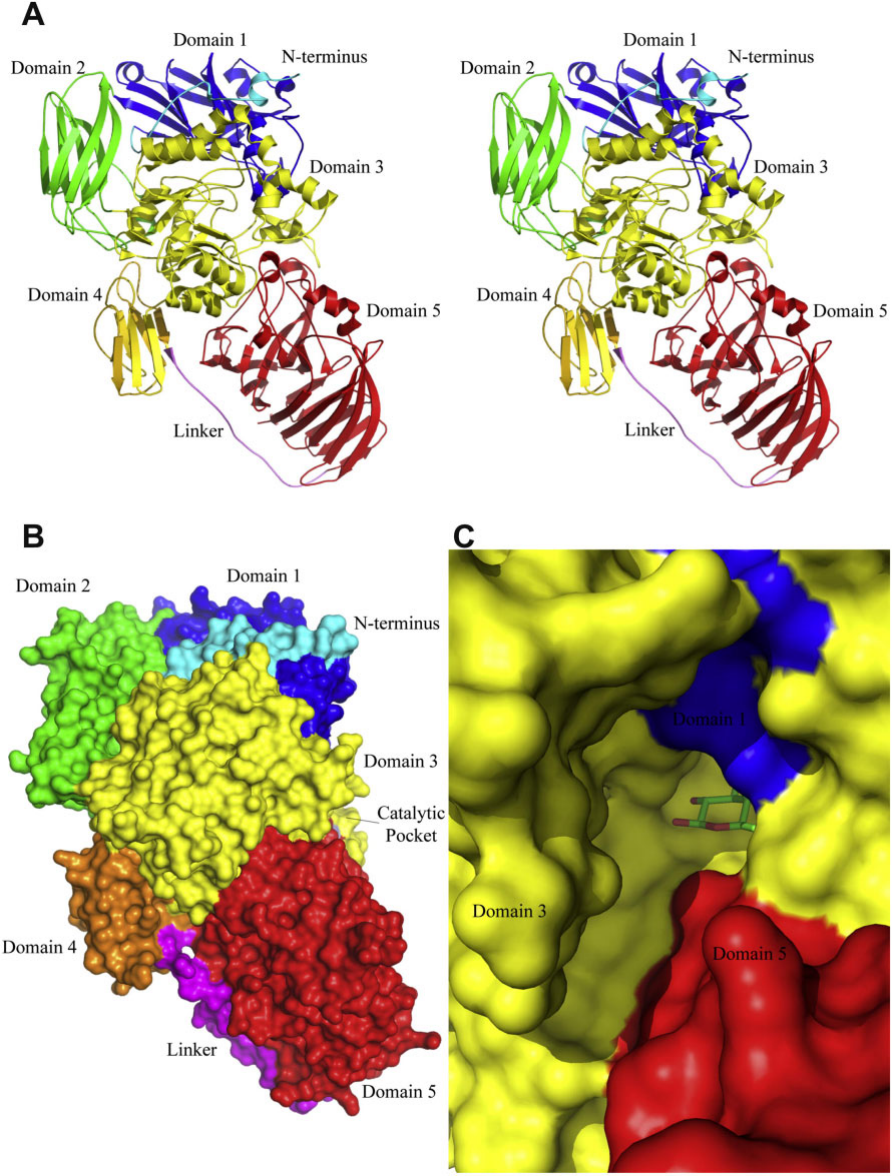
A. Stereo view of *Kluyveromyces lactis* β-galactosidase monomer in cartoon representation. Domains are represented in different colours. N-terminal region (cyan), domain 1 (blue), domain 2 (green), domain 3 (yellow), domain 4 (orange), linker (magenta) and domain 5 (red). B. Surface representation of the monomer with coloured domains following the same scheme. C. Zoomed view of the catalytic pocket entrance. Residues from domains 1, 5 and, mostly, 3 are building up the pocket entrance. A galactose bound to the active site is shown in stick representation. Reprinted with permission from Pereira-Rodríguez *et al*. Copyright (2012) Elsevier.

As an example, Fig. 1 shows the stereo view of the *K. lactis* β-galactosidase in its monomeric form, the surface representation, as well as the zoomed view of the active site pocket entrance. These authors elucidated that the monomer folds into five domains in a pattern conserved with other prokaryote enzymes solved for the GH family 2 (Pereira-Rodríguez *et al*., 2012). The active site of *K. lactis* β-galactosidase is build up mostly by residues from domain 3, which spans the residues 333–642, but some residues from domain 1 (Asn88, Val89, Asp187) and from domain 5 (Ala1000, Cys1001) also contribute to the narrow entrance that accesses the binding site. The most interesting fact in terms of oligosaccharide synthesis and specificity is the presence of two long insertions in domains 2 (residues 264–274) and 3 (residues 420–443) that shape the catalytic pocket and are unique as compared with other characterized β-galactosidases. Indeed, the unique insertion at loop 420–443 seems to play essential roles in ligand binding and recognition of the lactose molecules, as well as in selecting different acceptor molecules during transgalactosylation (Pereira-Rodríguez *et al*., 2012). These structural features may explain the high affinity of this enzyme for lactose and, therefore, the high hydrolytic activity against this substrate and the production of oligosaccharides derived from lactose in high amounts by transgalactosylation (Martínez-Villaluenga *et al*., 2008a).

**Fig. 2:**
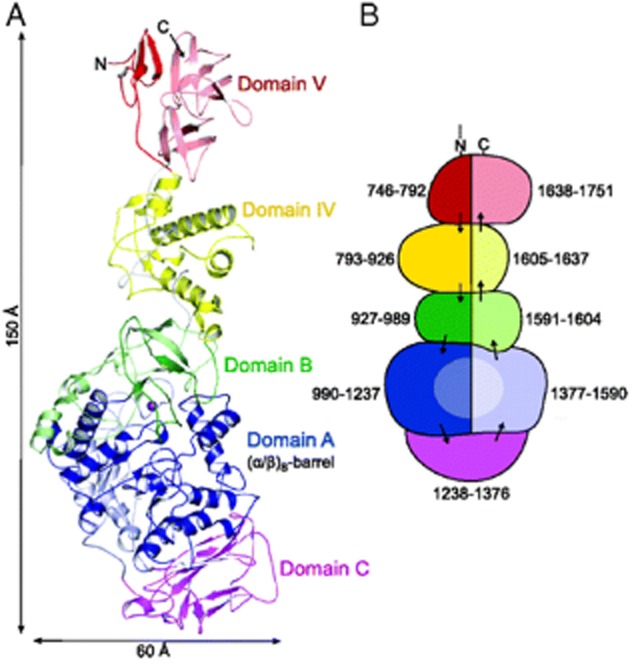
Overall structure of *Lactobacillus reuteri* 180 GTF180-ΔN glucansucrase.A. Crystal structure of GTF180-ΔN, the N- and the C-terminal ends of the polypeptide chain are indicated, the Ca^2+^ ion is shown as a magenta sphere.B. Schematic presentation of the ‘U-shaped’ course of the polypeptide chain. Domains A, B, C, IV and V are coloured in blue, green, magenta, yellow and red, respectively, with dark and light colours for the N- and C-terminal stretches of the peptide chain. Reprinted with permission from Vujičić-Žagar *et al*. Copyright (2010) The National Academy of Sciences.

On the other hand, there is more limited information about the three-dimensional structure of microbial glycansucrase enzymes. To date, only four structures of glucansucrases belonging to the GH family 70 have been obtained, including those from *Lactobacillus reuteri* 180 (Vujičić-Žagar *et al*., 2010), *L. reuteri* 121 (Pijning *et al*., 2012), *Streptococcus mutans* (Ito *et al*., 2011) and *Leuconostoc mesenteroides* NRRL B-1299 (Brison *et al*., 2012) (see PDB accession codes in Table 2). In all cases, the three-dimensional structures were obtained by crystallization of the recombinant truncated forms of the enzymes, which means that crystal structures of complete glucansucrase enzymes have not yet been reported to date (Leemhuis *et al*., 2013). These four enzymes present different linkage specificity, i.e. α(1→6), α(1→3), α(1→2), but share a common U-type fold, organized into five domains (A, B, C, IV and V). All the domains except domain C are made up from discontinuous segments of the polypeptide chain (Fig. 2). The domain A comprises a (α/β)_8_ barrel and harbours the catalytic site. In a similar way to GH13 α-amylases, the most important catalytic residues in glucansucrases are a nucleophile (Asp), a general acid/base (Glu) and a transition-state stabilizer (Asp), which lie at the bottom of a deep pocket in domain A (Leemhuis *et al*., 2013). Additionally, crystal structures combined with site-directed mutagenesis studies revealed that GH70 glucansucrases possess only one active site and have only one nucleophilic residue (Vujičić-Žagar *et al*., 2010), in contrast to the two-nucleophile mechanism proposed previously by Robyt and colleagues (2008) for dextransucrase enzymes and the mechanism for dextran synthesis.

Regarding fructansucrases, to date, five three-dimensional structures of truncated forms of different bacterial sources have been reported as shown in Table 2. All these enzymes display a five-bladed β-propeller fold, with an active site interacting with the donor substrate sucrose located in a deep pocket at the central axis surrounded by several semiconserved amino acid residues. The bottom of this pocket is tailored for fructosyl binding (Pijning *et al*., 2011).

### Catalytic mechanism of GHs

In general terms, enzymatic hydrolysis of the glycosidic bond takes place via general acid catalysis that requires two critical residues: a proton donor and a nucleophile/base (Davies and Henrissat, 1995). In the case of GHs used for the synthesis of food bioactive oligosaccharides, the hydrolysis occurs through a mechanism that gives rise to an overall retention of the initial conformation on the anomeric carbon (Koshland, 1953). In retaining enzymes, the nucleophilic catalytic base is in close vicinity of the sugar anomeric carbon (Davies and Henrissat, 1995).

**Fig. 3:**
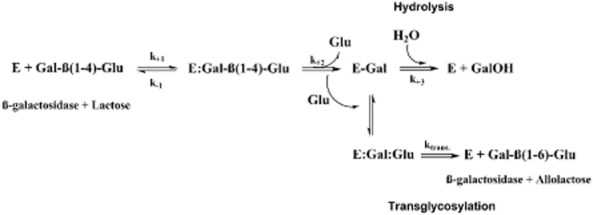
General scheme for the catalytic mechanism of β-galactosidase and a lactose molecule. Reprinted with permission from Brás *et al*. Copyright (2010) American Chemical Society.

The proposed general catalytic mechanism of β-galactosidase is illustrated in Fig. 3. Briefly, catalysis proceeds via a double-displacement reaction in which a covalent galactosyl-enzyme intermediate is formed and hydrolyzed via oxocarbenium ion-like transition states, while glucose is released (Withers, 2001). The active site contains two carboxylic acid residues, which have been identified specifically as Glu461 (proton-donor residue) and Glu537 (catalytic nucleophile group, forming the covalent galactosyl-enzyme intermediate) in the case of the β-galactosidase from *E. coli* (Juers *et al*., 2000). The second mechanistic step involves the attack of the covalent galactosyl-enzyme intermediate by a water molecule (hydrolysis) or a sugar molecule (transglycosylation), concomitantly with or followed by the transfer of a proton from a water/sugar to the proton donor, in a reverse mode of the first step (Brás *et al*., 2010) (Fig. 3).

**Fig. 4:**
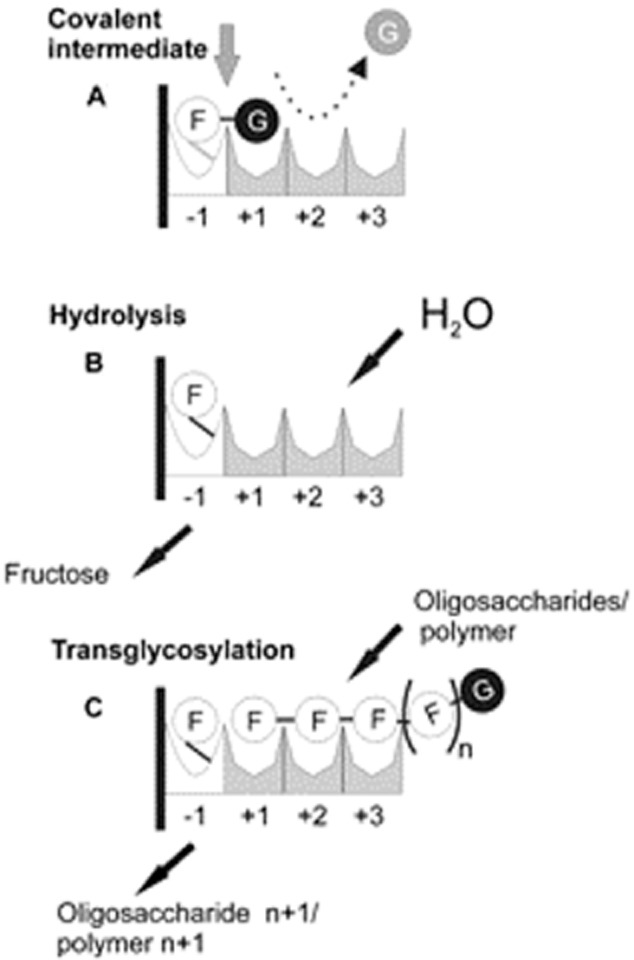
Schematic representation of the reaction sequences occurring in the active site of fructansucrase enzymes (FSs). The donor and acceptor subsites of FSs enzymes are mapped out based on the available three-dimensional structural information (Meng and Fütterer, 2003; Martínez-Fleites *et al*., 2005; Ozimek *et al*., 2006).A. Binding of sucrose to subsites −1 and +1 results in cleavage of the glycosidic bond (glucose released, shown in grey), and formation of a (putative) covalent intermediate at subsite −1 (indicated by a grey line). Depending on the acceptor substrate used, hydrolysis (by water) (B) or transglycosylation (C) reaction may occur [with oligosaccharides or the growing polymer chain, resulting in the synthesis of FOS (n + 1) or fructan polymer (n + 1), respectively]. Reprinted with permission from Ozimek *et al*. Copyright (2006) Society for General Microbiology.

As it could be expected, the catalytic mechanism of glycansucrases is also based on the double-displacement mechanism with retention of the anomeric configuration of the glucosyl/fructosyl moieties (van Hijum *et al*., 2006). First, the glycosidic linkage of the substrate is cleaved in the active centre of the enzyme between the amino acid residues located at the subsites −1 and +1 (according to the nomenclature proposed by Davies *et al*., 1997), resulting in a covalent glucosyl- (or fructosyl-) enzyme intermediate. In the second step, the glucosyl/fructosyl moiety is transferred to an acceptor with retention of the anomeric configuration (Leemhuis *et al*., 2013). Ozimek and colleagues (2006) illustrated the mode of action of fructansucrases based on the double-displacement reaction mechanism as it is shown in Fig. 4. The fructosyltransferase −1 subsite is highly specific for accommodating fructose units, whereas the +1 subsite seems to be more flexible, exhibiting affinity for both glucose (binding of sucrose, Fig. 4A) and fructose (binding sucrose as an acceptor substrate during transglycosylation, Fig. 4C). Consequently, sucrose enters the active site and occupies the −1 and +1 subsites, the glycosidic bond is cleaved as explained above, and a covalent fructosyl-enzyme intermediate is formed at −1 and glucose is released. Then, water may enter the active site, react with the fructosyl-enzyme intermediate, resulting in hydrolysis and release of fructose (Fig. 4B). Alternatively, a second acceptor substrate (sucrose) enters the active site, binds to the +1 and +2 subsites and reacts with the fructosyl-enzyme intermediate at −1, resulting in the oligosaccharide formation (i.e. fructo-oligosaccharides) (Fig. 4C). Further transglycosylation reaction may take place, resulting in chain elongation and polymer formation (Ozimek *et al*., 2006). Furthermore, it has been indicated that the amino acid residues responsible for the different product linkage type specificities of inulosucrase β(2→1) and levansucrase β(2→6) should orient the accepting fructosyl moiety at subsite +1 in quite different ways to form either of two types of glycosidic linkage (Pijning *et al*., 2011). These authors suggested that a non-conserved specific region of GH68 fructansucrases, i.e. the 1B–1C loop, could be involved in acceptor-binding interactions and determine the product specificity in bacterial fructansucrases.

## Enzymatic synthesis of novel lactose-derived oligosaccharides

Although the enzymatic synthesis of bioactive carbohydrates from lactose have been extensively investigated to produce GOS, recent research has focused on enzymatic transglycosylation using lactulose or mixtures of lactose with other carbohydrates, developing new bioactive oligosaccharides varying in their monosaccharide composition and structural features as it will be explained below. During enzymatic hydrolysis of lactose in the presence of other carbohydrates, they can act as acceptors in the galactosylation process to give new β-linked galactosyl oligosaccharides (Li *et al*., 2009; Schuster-Wolff-Bühring *et al*., 2009; Seki and Saito, 2012). Lactose-derived oligosaccharides can also be produced during enzymatic hydrolysis of other carbohydrates using lactose as acceptor (Table 1).

### Lactosucrose

Lactosucrose (*O*-β-D-galactopyranosyl-(1→4)-*O-*α-D-glucopyranosyl-(1→2)-β-D-fructofuranoside), also named lactosylfructoside, is a non-reducing trisaccharide obtained from a mixture of lactose (β-D-galactopyranosyl-(1→4)-α-D-glucose) and sucrose (α-D-glucopyranosyl-(1→2)-β-D-fructose) by enzymatic transglycosylation. Synthesis reaction may occur either by transfructosylation by β-fructofuranosidase (EC 3.2.1.26) from *Arthrobacter sp.* K-1 (Fujita *et al*., 1990; Mikuni *et al*., 2000; Pilgrim *et al*., 2001) or levansucrases (EC 2.4.1.10) from various bacteria strains such as *Bacillus subtilis* (Park *et al*., 2005)*, B. natto* (Takahama *et al*., 1991)*, Paenibacillus polymyxa* (Choi *et al*., 2004) and *Zymomonas mobilis* (Han *et al*., 2009), or through transgalactosylation by β-galactosidase (EC 3.2.1.23) from *B. circulans* (Li *et al*., 2009). In the first case (transfructosylation reaction), lactose acts as acceptor of the fructosyl moiety of sucrose whereas in the latter (transgalactosylation), lactose can act both as donor and acceptor so that a mixture of GOS are produced, in addition to lactosucrose, which is formed by transferring the galactosyl moiety from lactose to sucrose.

The first study reported in the literature on lactosucrose synthesis was undertaken by Avigad in 1957 who reported its production by levansucrase from *Aerobacter levanicum*. Subsequently, in the 1990's, lactosucrose industrial production was developed by Fujita and colleagues (1992). Since then, lactosucrose has been commercially produced by transglycosylation for over 20 years (Mu *et al*., 2013). It is used as a low-calorie sweetener because of being hardly hydrolyzed by human digestive enzymes or prebiotic ingredient because of its ability to enhance the growth of beneficial bifidobacteria and to maintain the balance of the intestinal environment (Yoneyama *et al*., 1992; Ogata *et al*., 1993; Ohkusa *et al*., 1995). Other healthy effects that have been linked to the ingestion of lactosucrose are the prevention of constipation and reduction of serum hyperlipidemia (Kitahata and Fujita, 1993; Côté, 2007).

Lactosucrose marketing has received particular interest in Japan where over 30 food products containing this prebiotic ingredient have been certified as food for specified health use, being Ensuiko Sugar Refining and Hayashibara Shoji the two major manufacturers. Besides Japan, lactosucrose is also marketed in the United States followed by Europe as it can be considered an emerging prebiotic (Crittenden, 2006; Rastall, 2010).

### Galactooligosaccharides derived from lactulose (GOS-Lu)

Typical GOS comprises a complex mixture of carbohydrates obtained by transgalactosylation reactions from lactose using β-galactosidases of different origins. This complexity relies on a different chain length (normally from disaccharides to octasaccharides) and on the type of glycosidic linkages. The composition of GOS is normally highly affected by several factors including the enzyme source, lactose concentration, substrate composition and reaction conditions (temperature, pH, time and enzyme charge). Using lactose as a single substrate, the galactose released during enzymatic hydrolysis of lactose may be transferred to another lactose molecule, being linked to the galactose moiety by β(1→6), β(1→4) or β(1→3) glycosidic bonds, depending on the enzyme source. The trisaccharides formed may be elongated by new linked galactosyl moieties (Gänzle, 2012). The galactosyl residue may also be transferred to the glucose released to give allolactose (Mahoney, 1998).

In a series of pioneering works (Cardelle-Cobas *et al*., 2008; Martínez-Villaluenga *et al*., 2008b), the use of lactulose (4-*O*-β-D-galactopyranosyl-D-fructose) instead of lactose as precursor of novel GOS was proposed. The interest of this type of oligosaccharides could rely on the multiple beneficial properties that lactulose exhibits *per se*, and consequently, GOS produced from the straight transglycosylation of lactulose instead of lactose might possess an added value as compared to the conventional GOS. In this sense, lactulose is a synthetic disaccharide commercially produced by chemical isomerization of lactose under basic media (Mendez and Olano, 1979) and is recognized since the 1950's as a bifidogenic factor when added to the diet (Petuely, 1957). Lactulose is also efficient in the treatment of chronic constipation (Mayerhofer and Petuely, 1959) and to control portal-systemic encephalopathy (Bircher *et al*., 1966), a chronic disease state that caused elevated blood ammonia levels that interferes with brain functions, causing cognitive dysfunction and psychiatric disorders. Lactulose helps restore normal neurological function when fermented by colonic bacteria to short chain fatty acids that acidify the content of the large bowel resulting in the trapping and reabsorption of ammonia. Today, lactulose is used in medicine as well as in the food industry (Schumann, 2002).

**Fig. 5:**
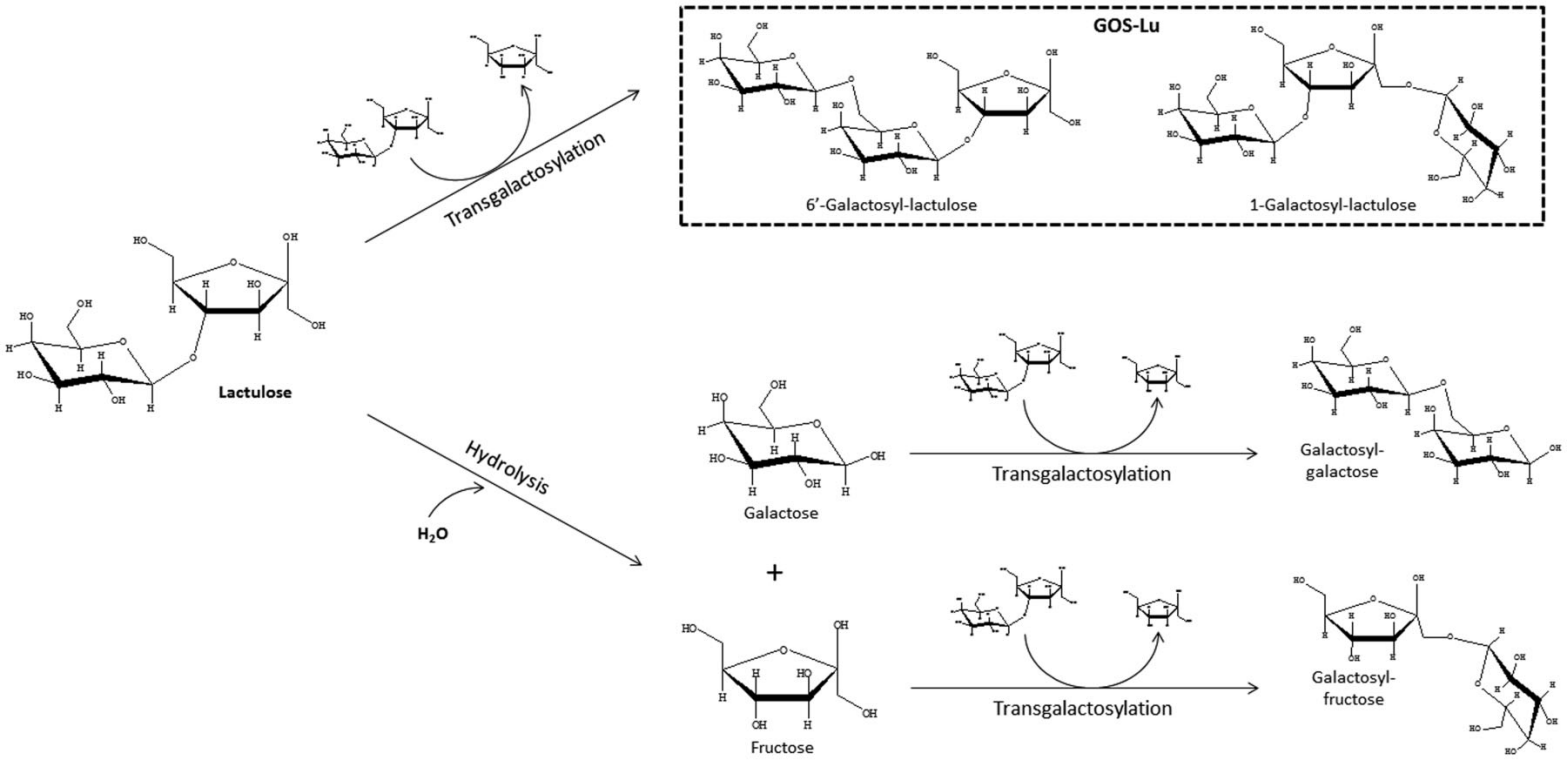
General scheme of transgalactosylation and hydrolytic processes involving lactulose and β-galactosidase to produce GOS-Lu, galactobioses and galactosyl-fructoses.

Lactulose has indeed been demonstrated to be hydrolyzed by the action of β-galactosidases from different microbial origin, such as *Kluyveromyces lactis* (Martínez-Villaluenga *et al*., 2008b; Cardelle-Cobas *et al*., 2011a), *Aspergillus aculeatus* (Cardelle-Cobas *et al*., 2008), *Aspergillus oryzae* (Hernández-Hernández *et al*., 2011) and, albeit at a much lesser extent, *Bacillus circulans* (Corzo-Martínez *et al*., 2013). Consequently, lactulose can be used as a substrate for the efficient oligosaccharide synthesis during transglycosylation because the galactose released is transferred to another lactulose molecule leading to the formation of GOS-Lu, as well as to galactose and fructose monomers derived from the lactulose hydrolysis to give different galactobioses and galactosyl-fructoses as displayed in Fig. 5.

Similarly to GOS-derived from lactose, a complex mixture of GOS-Lu with different glycosidic linkages and molecular weights is obtained and the yield is affected by the ratio of hydrolytic and transferase activities of the enzyme. In a similar way to conventional GOS, this ratio depends mainly on the enzyme source, the concentration of initial lactulose and the reaction conditions (temperature, pH, time and enzyme charge). In general terms, the transferase activity is favoured at high initial lactulose concentration, elevated reaction temperature and lower water activity (Tzortzis and Vulevic, 2009), providing good yields in GOS-Lu. Table 3 summarizes the characterized carbohydrate composition of GOS-Lu resulting from the reaction mixtures using different enzymes, as well as the optimum conditions leading to the maximum levels of GOS-Lu. Optimum pH and temperature conditions are constrained by the source of enzyme, as well as the type of glycoside bond formed between the transferred galactose moieties and lactulose, galactose or fructose to give the different oligosaccharides. Concretely, β-galactosidases from *Aspergillus* sp. predominantly produced oligosaccharides with β(1→6) linkages, such as 6′-galactosyl-lactulose and, in lesser concentrations, 6-galactobiose and allolactulose, while β-galactosidase from *K. lactis* synthesized similar levels of 6′-galactosyl-lactulose and 1-galactosyl-lactulose (Table 3). In contrast, the main trisaccharide formed after the synthesis with a β-galactosidase from *B. circulans* was the 4′-galactosyl-lactulose, suggesting the preferential formation of β(1→4) glycosidic linkages, in good agreement with the findings described for the synthesis of GOS from the transgalactosylation of lactose (Rodríguez-Colinas *et al*., 2012).

**Table 3:** Oligosaccharide composition and yield of GOS-Lu using different microbial β-galactosidases

β-Galactosidase source	Optimum reaction conditions	Oligosaccharide composition	Total GOS-Lu yield	Main glycosidic linkage
*Kluyveromyces lactis*^a^	250 g l^−1^ Lactulose; 50°C; pH 7.5, 2 h	31% Monosaccharides	27%	β(1→6) β(1→1)
		42% Lactulose		
		10% 6′-Galactosyl-lactulose		
		11% 1-Galactosyl-lactulose		
		6% Unidentified oligosaccharides		
*Aspergillus aculeatus*^a^	450 g l^−1^ Lactulose; 60°C; pH 6.5, 7 h	35% Monosaccharides	29%	β(1→6)
		36% Lactulose		
		15% 6′-Galactosyl-lactulose		
		2% 1-Galactosyl-lactulose		
		12% Unidentified oligosaccharides		
*Aspergillus oryzae*^b^	450 g l^−1^ Lactulose; 50°C; pH 6.5, 1 h	30% Monosaccharides	50%	β(1→6)
		20% Lactulose		
		20% 6′-Galactosyl-lactulose		
		3% 6-Galactobiose		
		1% Allolactulose		
		26% Unidentified oligosaccharides		
*Bacillus circulans*^c^	300 g l^−1^ Lactulose; 50°C; pH 6.5, 5 h	4′-Galactosyl-lactulose^d^	–d	β(1→4)

a. Data compiled from Cardelle-Cobas *et al*., 2012.

b. Data compiled from Cardelle-Cobas, 2009.

c. Data compiled from Corzo-Martínez *et al*., 2013.

d. Qualitative determination (no quantitative data available).

Concerning GOS-Lu yields, although the quantification described in Table 3 was carried out from di-, tri- and tetrasaccharides, evidences on presence of penta- and hexasaccharides in minor amounts have been also reported (Hernández-Hernández *et al*., 2011). Thus, considering disaccharides (not including lactulose), tri- and tetrasaccharides, GOS-Lu yields ranged from 27% to 50%, with the β-galactosidase from *A. oryzae* providing the highest yield, whereas those of *K. lactis* and *A. aculeatus* showed lower transglycosylation activity (Table 3). β-Galactosidase from *B. circulans* showed an extremely low hydrolytic activity on lactulose and, therefore, only low levels of GOS-Lu could be determined (Corzo-Martínez *et al*., 2013).

Up to now, there are considerable *in vitro* and *in vivo* data supporting some remarkable beneficial effects of GOS-Lu, specially, on the gastrointestinal system. Thus, a preliminary *in vitro* study carried out with pure cultures of different probiotic bacteria showed that this type of oligosaccharide was fermented by different strain of *Bifidobacterium*, *Lactobacillus* and *Streptococcus* (Cardelle-Cobas *et al*., 2011b). Later on, *in vitro* pH-controlled fermentation studies performed with human faecal samples demonstrated the bifidogenic activity of GOS-Lu (Cardelle-Cobas *et al*., 2009; 2012). Likewise, *in vivo* studies carried out with growing rats revealed a higher resistance of GOS-Lu to gastrointestinal digestion and absorption in the small intestine than that of GOS from lactose, which was attributed to the great resistance of galactosyl-fructoses to the hydrolytic action of mammalian digestive enzymes. These results highlight the key role played by the monomer type and glycosidic linkage involved in the oligosaccharide structure. In addition, the bifidogenic effect observed in *in vitro* studies was also corroborated by this *in vivo* assay with rats fed 1% *(w/w)* of GOS-Lu for 14 days (Hernández-Hernández *et al*., 2012). Furthermore, a significant and selective increase of *Bifidobacterium animalis* counts in the caecum and colon section of these rats was observed (Marín-Manzano *et al*., 2013). Taken together, these data clearly support the potential use of GOS-Lu as novel prebiotic ingredients as meet the three criteria that a food ingredient must satisfy to be considered as prebiotic: (i) non-digestibility, (ii) fermentation by the intestinal microbiota and (iii) a selective stimulation of the growth and/or activity of those intestinal bacteria that contribute to health and well-being. Nevertheless, because clinical trials are essential to make any claim of prebiotic properties, *in vivo* studies carried out with humans should be performed in order to definitively establish their prebiotic properties.

On the other hand, GOS-Lu has been also reported to exhibit *in vitro* immunomodulatory properties using intestinal (Caco-2) cells (Laparra *et al*., 2013). GOS-Lu inhibited the production of pro-inflammatory factors, such as necrosis factor-alpha and interleukin-1 beta, by intestinal cells stimulated by the pathogen *Listeria monocytogenes* CECT 935. The authors suggested that GOS-Lu had the ability to impair the interaction of virulence factors of this intestinal pathogen with the epithelial cells.

Another beneficial activity associated to prebiotics is the increase in absorption of several minerals, such as calcium or magnesium, across the epithelial cells of colon and caecum (Gibson *et al*., 2004). In this context, GOS-Lu has been recently shown to improve iron absorption in an *in vivo* iron-deficient animal model carried out with Wistar rats (J.M. Laparra, M. Díez-Municio, M. Herrero and F.J. Moreno, unpublished).

### Lactulosucrose

**Fig. 6:**
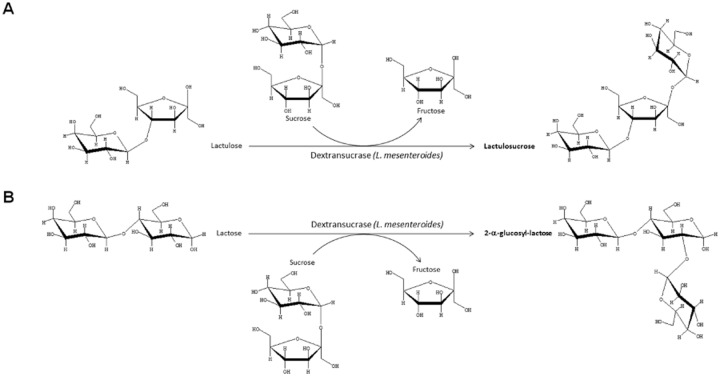
Process scheme for the synthesis of (A) lactulosucrose and (B) 2-α-glucosyl-lactose by transglucosylation of lactulose and lactose, respectively, catalyzed by a dextransucrase from *Leuconostoc mesenteroides* B-512F.

Lactulose may also be used as glycosyl acceptor during enzymatic hydrolysis of other carbohydrates. This is of particular interest because it can expand the opportunities for the synthesis of new lactulose-derived oligosaccharides with novel structures and improved prebiotic properties. This approach has been used to prepare lactulose derivatives as lactulosucrose (β-D-galactopyranosyl-(1→4)-β-D-fructofuranosyl-(2→1)-α-D-glucopyranoside), a trisaccharide produced from the transfer of the glucosyl moiety of sucrose to the C-2 group of the reducing end unit of lactulose and catalyzed by a dextransucrase produced by *Leuconostoc mesenteroides* B-512F (Fig. 6A) (Díez-Municio *et al*., 2012a). Among the reaction parameters optimized, the concentration ratio of donor (sucrose) to acceptor (lactulose) and the dextransucrase charge had a considerable impact on the yield of lactulosucrose. From the reported results, it could be concluded that using the same concentration of dextransucrase, the higher the ratio of lactulose to sucrose, the higher the amount of synthesized lactulosucrose. Furthermore, in general terms, as the concentration of enzyme was increased, the levels of lactulosucrose were higher and achieved at shorter reaction times. The enzymatic reaction carried out under optimal conditions, i.e. at 30°C, pH 5.2 with a dextransucrase charge of 2.4 U ml^−1^ and a starting concentration of sucrose:lactulose 30:30 (expressed in g 100 ml^−1^) led to the production of lactulosucrose with a high yield of 35% in weight respect to the initial amount of lactulose. Likewise, this enzymatic reaction was also characterized by a high regioselectivity, which allowed the major formation of lactulosucrose, and only traces of other acceptor-reaction products susceptible to correspond to lactulose-based tetra- and pentasaccharide could be detected. This non-complex oligosaccharide pattern makes easier the isolation, as well as the potential scale-up of the manufacture process.

Concerning bioactive properties, preliminary data on selective fermentation of lactulosucrose by pure cultures of different probiotic bacteria have revealed a significant bifidogenic effect. In fact, three different bifidobacterial strains, *B. lactis* BB-12, *B. breve* 26 M2 and *B. bifidum* HDD541, grew better on lactulosucrose than on lactulose or lactosucrose (T. García-Cayuela, M. Díez-Municio, M. Herrero, M.C. Martínez-Cuesta, C. Peláez, T. Requena and F.J. Moreno, unpublished). In addition, having lactulose as a core structure, lactulosucrose might possess the beneficial properties attributed to lactulose, e.g. prebiotic activity, enhancement of mineral absorption, blood glucose-lowering effects and reduction in intestinal transit time, among others (Schumann, 2002). Also, lactulosucrose might possess lower fermentation rates as compared with lactulose because longer carbohydrate chains are normally fermented slower (Perrin *et al*., 2002). This would increase its interest as prebiotic by increasing its capacity to reach the distal parts of the colon, where many chronic gut disorders take place (Gibson *et al*., 2004). In summary, lactulosucrose could be an emerging bioactive oligosaccharide with interesting food and/or pharmaceutical applications.

### 2-α-Glucosyl-lactose

Similarly to the enzymatic synthesis of lactulosucrose, lactose has also proven to be an efficient acceptor in the *L. mesenteroides* B-512F dextransucrase-catalyzed reaction using sucrose as a donor. This enzyme catalyzes the transfer of glucose released from the hydrolysis of sucrose to the 2-hydroxyl group of the reducing glucose unit of lactose to form the trisaccharide 2-α-glucosyl-lactose (Fig. 6B) (Díez-Municio *et al*., 2012b). In this case, the effect of synthesis conditions, including concentration of substrates, molar ratio of donor/acceptor, enzyme concentration, reaction time and temperature were also evaluated and optimized. This trisaccharide was produced not only from buffered lactose solution, but also from several industrial cheese whey permeates. In all cases, 2-α-glucosyl-lactose was produced with high regioselectivity and excellent yields, reaching values up to 47% (when using lactose solutions) and 52%, in weight respect to the initial amount of lactose, when cheese whey permeate was used as starting material. It was hypothesized that the mineral composition contained in the studied cheese whey permeates (including the trace minerals) might have an effect on the transfer rate of the dextransucrase and, therefore, on the content of 2-α-glucosyl-lactose, in a similar way to the effect of different cations, such as sodium, potassium and magnesium had on the transgalactosylation activity of several fungal and bacterial β-galactosidases (Garman *et al*., 1996; Montilla *et al*., 2012).

According to the elucidated structure (Fig. 6B), this trisaccharide could also be a suitable candidate for a new prebiotic ingredient because the reported high resistance of α(1→2) linkages to the digestive enzymes in humans and animals (Valette *et al*., 1993; Remaud-Simeon *et al*., 2000) as well as to its potential selective stimulation of beneficial bacteria in the large intestine mainly attributed to the two linked glucose units located at the reducing end that reflects the disaccharide kojibiose (2-α-D-glucopyranosyl-D-glucose) (Nakada *et al*., 2003; Sanz *et al*., 2005). In fact, 2-α-glucosyl-lactose has been recently used as an efficient precursor for the enzymatic synthesis of kojibiose following its hydrolysis with a β-galactosidase from *K. lactis* and further purification (Díez-Municio *et al*., 2013a; 2014). Kojibiose is a naturally occurring disaccharide present in honey, beer or sake, although at low levels, which makes difficult its isolation from natural sources at high scale. Apart from the prebiotic properties attributed to kojibiose, important potential pharmaceutical applications have been also linked to this particular disaccharide (Srivastava *et al*., 2011).

## Conclusions

The development of simple and convenient methods for the efficient synthesis of bioactive oligosaccharides offers opportunities to facilitate their use as functional ingredients. In general, the use of microbial glycoside hydrolase enzymes have a great potential in the food carbohydrate field, as they normally exhibit high substrate specificity, regio- and stereospecificity, and can be economically produced. This review has addressed the suitability of lactose in the presence or absence of sucrose to act as precursor for the high-yield synthesis of novel and potentially functional dietary oligosaccharides. Lactose, sucrose and their corresponding agro-industrial by-products containing these disaccharides, such as beet and cane molasses or cheese whey permeate, share the characteristic of being derived from readily available and inexpensive raw materials. These facts could contribute to the development of efficient and inexpensive manufacturing process of promising functional oligosaccharides, which could exert a series of beneficial properties on host health.

Lastly, engineering selected enzymes by site-directed mutagenesis studies could provide new mutants with different specificity (e.g. improved transglycosylation activities) and, thus, guide the transglycosylation reaction towards the synthesis of tailor-made oligosaccharides of specific interest for human health. The search of new microbial enzyme sources could also contribute to the production of novel oligosaccharides with new and/or improved bioactive and physico-chemical properties.
